# The correlation of serum long non‐coding RNA ANRIL with risk factors, functional outcome, and prognosis in atrial fibrillation patients with ischemic stroke

**DOI:** 10.1002/jcla.23352

**Published:** 2020-05-01

**Authors:** Weixian Zeng, Jun Jin

**Affiliations:** ^1^ Department of Intensive Care Unit Shenzhen Hospital of Southern Medical University Shenzhen China; ^2^ Adult Intensive Care Unit The University of Hong Kong‐Shenzhen Hospital Shenzhen China

**Keywords:** antisense non‐coding RNA in the INK4 locus, atrial fibrillation, ischemic stroke, long non‐coding RNA

## Abstract

**Background:**

This study aimed to evaluate the predictive value of long non‐coding RNA (lncRNA) antisense non‐coding RNA in the INK4 locus (ANRIL) for atrial fibrillation (AF) patients with ischemic stroke and investigate its correlation with risk factors, functional outcome, and prognosis.

**Methods:**

A total of 386 consecutive AF patients were recruited. AF patients were followed up for 24‐48 months by outpatient follow‐up, telephone follow‐up, and medical record. The time of ischemic stroke in patients with AF was recorded, and follow‐up was continued for 6 months. LncRNA ANRIL expression from serum was detected by quantitative real‐time polymerase chain reaction (qRT‐PCR).

**Results:**

Compared with the AF with ischemic stroke group (14.3 ± 2.3), patients in the AF without ischemic stroke group (11.9 ± 1.8) had significantly lower serum lncRNA ANRIL levels (*P* < .05). The sensitivity and specificity of lncRNA ANRIL for identifying AF with ischemic stroke were 76.6% and 81.4%, respectively. Spearman correlation analysis results shown that lncRNA ANRIL was significantly correlated with the NIHSS score (*r*_Spearman_ = .490, *P* < .001) and the mRS score (*r*_Spearman_ = .466, *P* < .001). Compared with the lncRNA ANRIL high‐expression group, the recurrence‐free survival (RFS) of the lncRNA ANRIL low‐expression group was significantly higher (*χ^2^* = 11.009, log‐rank *P* < .001). Cox proportional regression model analysis indicated that the serum lncRNA ANRIL level (*P* = .004), NIHSS score (*P* = .001), infarct volume (*P* = .035), and smoking (*P* < .001) were the risk factors for AF with ischemic stroke.

**Conclusion:**

Serum lncRNA ANRIL exerts a good predictive value for AF with ischemic stroke, and its increased expression is correlated with worse RFS for patients.

## 1 INTRODUCTION

Atrial fibrillation (AF) is the most common persistent arrhythmia.^1^ A multi‐center study published in 2013 analyzed AF epidemiological data in 10 different regions (4 towns and 6 rural areas) and shown that the prevalence of AF in people aged 35‐59 years was 0.42%, and that of those over 60 years old was 1.83%.^2^ Among people over 80 years old, the incidence of AF was up to 7.5%.^2^ During the onset of AF, the frequency of atrial agitation can reach 300‐600 beats per minute, which causes the patient's atrial to lose effective contractile function.^3^ The oxidative damage and thrombosis of AF are hot spots in recent years. It involves a variety of oxidative stress injuries including lymphocyte DNA damage and myeloperoxidase, which leads to damage to the brain, heart, kidney, and other organs.^4^, ^5^, ^6^ Among them, ischemic stroke is one of the greatest complications of AF, and the consequences of ischemic stroke caused by AF are more serious, and the mortality rate is extremely high.^7^, ^8^

Long non‐coding RNAs (lncRNAs) are a class of sequences longer than 200 nucleotides that were once considered as junk sequences.^9^ The mechanism of lncRNA mainly includes the regulation of transcription and post‐transcription.^10^, ^11^ Recent studies have shown that lncRNA not only affects the progress and outcome of ischemic stroke through ischemia‐reperfusion injury, inflammatory response, angiogenesis, nerve repair, and other pathways, and its early diagnosis, disease classification and prognosis assessment are also important.^12^, ^13^, ^14^

LncRNA antisense non‐coding RNA in the INK4 locus (ANRIL) is the gene most closely linked to atherosclerosis, which is significantly related to the occurrence of cerebral ischemia.^15^ In a prospective case‐control study, Zhang et al^16^ analyzed 724 patients with atherosclerotic thrombosis, 466 patients with lacunar infarction, 462 patients with cerebral hemorrhage, and an equivalent proportion of 1664 normal people. During a 4.5‐year follow‐up, they found that patients with lncRNA ANRIL variant rsl0757278 had a 1.47‐ and 1.60‐fold higher risk of cerebral thrombosis and cerebral hemorrhage. In addition, Bai et al^17^ applied Chinese Han umbilical vein endothelial cells and HepG2 cells to find that lncRNA ANRIL may bind to chromatin through Alu repeats, thereby regulating the expression of rs2043211 related to ischemic stroke. All these studies suggest that lncRNA ANRIL is involved in the process of ischemic stroke. The purpose of this study was to investigate the correlation of serum lncRNA ANRIL in AF with ischemic stroke and to provide a new way for the clinical diagnosis and treatment of AF with ischemic stroke.

## 2 MATERIALS AND METHODS

### 2.1 Number of subjects required for current study

The formula for calculating the number of diagnostic test samples is as follows: $n={\left(\mu_{\alpha }^{2}/\delta \right)}^{2}\left(1-P\right)P,$ where *α* is set to 0.05, μ_α_ is set to 1.96, *δ* is set to .05, and the *P*_Sensitivity_ is set to 90%. After substituting μ_α_ = 1.96, *δ* = .10, and *P*_Sensitivity_ = 90% into the formula, the number of cases that can be obtained is 130; that is, at least 130 patients are required to obtain reliable statistical results in our study.

### 2.2 Inclusion of study subjects

In this study, patients who did not complete follow‐up or were lost to follow‐up were excluded (n = 93). In the end, a total of 386 consecutive AF patients who went to the outpatient clinic of The University of Hong Kong‐Shenzhen Hospital from January 2016 to December 2017 were selected. The diagnosis of each AF patient was based on the electrocardiogram by two physicians. The characteristics of electrocardiogram in patients with AF mainly include the following aspects: (a) The P wave disappears and is replaced by a series of fast, varying size, and different forms of AF waves (F wave), with a frequency of about 350‐600 beats per minute. (b) The RR interval is irregular. (c) The shape of the QRS wave is usually normal. When the ventricular rate is too fast, differential conduction in the ventricle occurs, and the QRS wave widens and deforms. All AF patients denied having a history of stroke. AF patients were followed up for 24‐48 months after outpatient treatment. The follow‐up methods were mainly based on outpatient follow‐up, telephone follow‐up, and medical record. The time of ischemic stroke in patients with AF was recorded, and follow‐up was continued for 6 months from the onset of ischemic stroke. The patients developing stroke within 24‐48 months were assigned as AF with ischemic stroke, and the functional prognosis, recurrence‐free survival, and survival status were recorded. The acquisition of blood samples and clinical data of this study were approved by the Ethics Committee of The University of Hong Kong‐Shenzhen Hospital ethics committee (LH‐S2015073).

### 2.3 Blood samples and basic parameters collection

In the first outpatient visit of 386 patients with AF, 2 mL of peripheral venous blood was collected from each patient using blood collection tubes containing coagulant, and then, the serum was separated and stored in a −80° ultra‐low‐temperature refrigerator for later use. Moreover, patients' age, gender, body mass index (BMI), hypertension, diabetes, drinking, smoking, blood lipid levels, procalcitonin (PCT), high‐sensitivity C‐reactive protein (hs‐CRP), fibrinogen, D‐dimer, family history of ischemic stroke, medications, etc, were collected.

All patients with AF were scored according to the National Institutes of Health Stroke Scale (NIHSS) immediately after the occurrence of ischemic stroke, and the infarct volume was measured by MRI within 24 hours of onset.

### 2.4 Functional outcome and prognosis evaluation of AF with ischemic stroke at 6 months of follow‐up

Functional outcomes, recurrence‐free survival, and survival status were assessed at 6 months after ischemic stroke in patients with AF. Functional outcome was evaluated based on a modified Rankin Scale (mRS). An mRS score between 0 and 2 indicates that the patient's functional recovery is good (favorable functional outcome), while an mRS score of 3‐6 or death is a poor function recovery (unfavorable functional outcome). In addition, the time to recurrence of ischemic stroke during the 6‐month period was recorded.

### 2.5 Serum total RNA extraction and quantitative real‐time polymerase chain reaction (qRT‐PCR) detection

Serum total RNA was extracted according to the steps of the serum RNA extraction kit (QIAGEN, Lot number: 50504) and then reverse‐transcribed into cDNA using the genome‐free‐reverse transcription kit (Lot number: RX180417A). QRT‐PCR was performed according to SYBR GREEN instructions (Lot number: 190 312). LncRNA ANRIL and glyceraldehyde‐3‐phosphate dehydrogenase (GAPDH) primer sequences are shown in Table 1. The relative expression of lncRNA ANRIL was calculated using the 2^−Δ^ *^C^* ^t^ method. In this study, the amplification efficiencies of lncRNA ANRIL and GAPDH were 97.9% and 98.4%, respectively.

**Table 1:** Primer sequences of lncRNA ANRIL and GAPDH

RNA	Primer sequence (5′‐3′)
LncRNA ANRIL upstream	ACAACGGCATTCGCGGACAGGCGA
LncRNA ANRIL downstream	ATTTCCAACCGGAGGTCGCCACATT
GAPDH upstream	AGAAGGCTGCCTGCAGGCGCTCATTTG
GAPDH downstream	GCAGGAGGCGATGGCAGCTGATGAT

### 2.6 Statistical analysis

Statistical analysis was performed using SPSS 20.0. Comparisons between groups of normal distribution data (mean ± SD) were performed using *t* test. Spearman test was used to calculate the correlation between lncRNA ANRIL and NIHSS/mRS. Receiver operator characteristic curve (ROC) analysis calculated the area under ROC curve (AUC) of the lncRNA ANRIL level to discriminate AF with ischemic stroke. The Kaplan‐Meier method was used to calculate the survival analysis of serum lncRNA ANRIL on AF with ischemic stroke, and the Cox proportional regression model was used to analyze the potential factors affecting the prognosis of AF with ischemic stroke. The inspection level *α* in this study was .05.

## 3 RESULTS

### 3.1 Comparison of basic information and lncRNA ANRIL expressions between AF with ischemic stroke group and AF without ischemic stroke group

**Figure 1:**
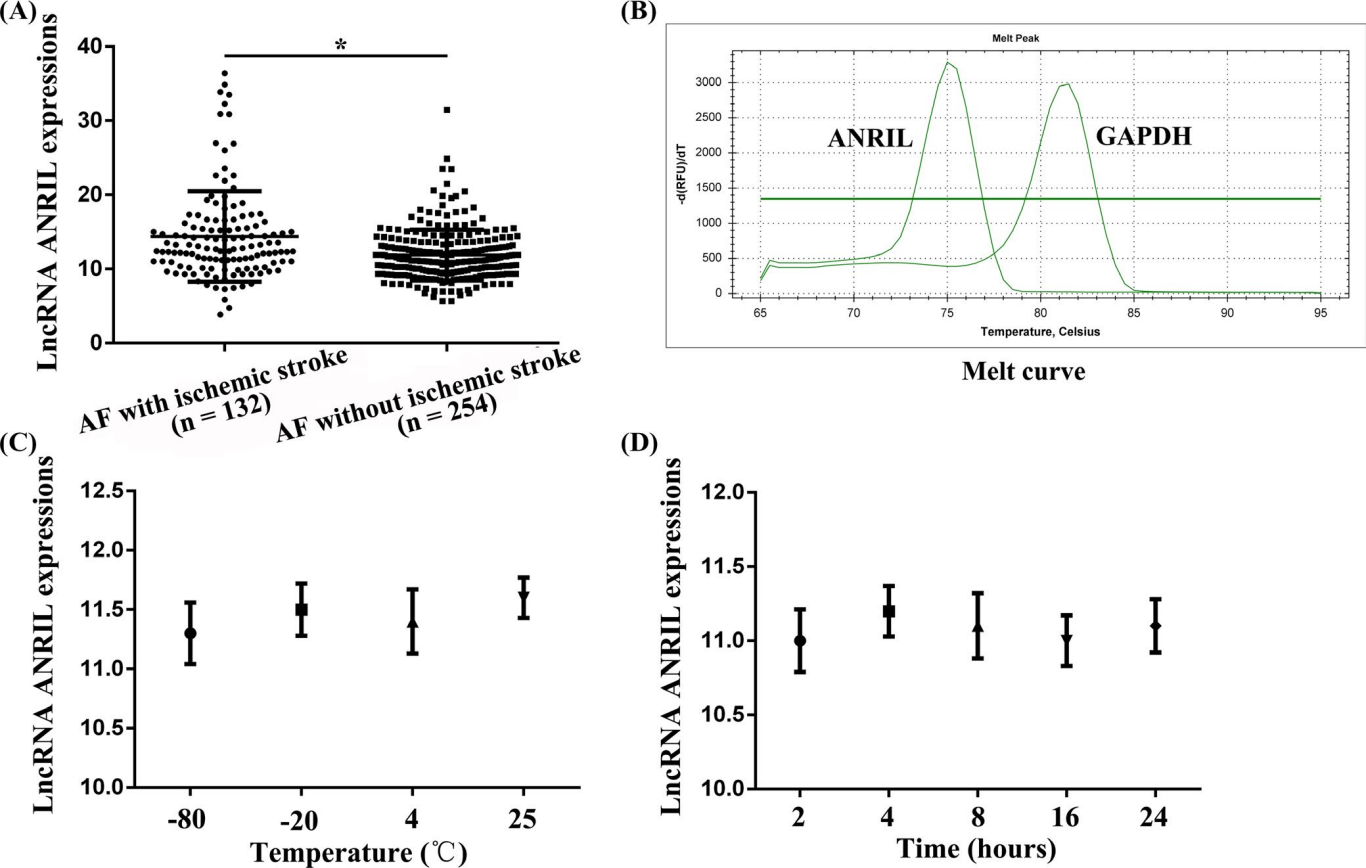
Detection of serum LncRNA ANRIL expression. A, Compared with patients in the AF with ischemic stroke group, patients in the AF without ischemic stroke group had significantly lower serum lncRNA ANRIL levels. B, The qRT‐PCR amplification melt curves of lncRNA ANRIL and GAPDH. C, Effects of different temperatures on lncRNA ANRIL expression. D, Effects of different in vitro time on ANRIL expression. *P < .05

Of the 386 patients with AF, a total of 132 (34.2%) patients had ischemic stroke. Therefore, patients were divided into AF with ischemic stroke group (n = 132) and AF without ischemic stroke group (n = 254). The basic information of patients is shown in Table 2. Compared with the AF with ischemic stroke group (14.3 ± 2.3), AF without ischemic stroke (11.9 ± 1.8) had significantly lower serum lncRNA ANRIL levels (*P* < .05, Figure 1A and Table 2). The qRT‐PCR amplification melt curve showed a single amplification product, suggesting high amplification specificity (Figure 1B). The stability test results are shown in Figure 1C,D. Serum lncRNA ANRIL expression was not affected by blood ex vivo time and storage temperature. In addition, we also found that the number of patients with smoking history in the AF with ischemic stroke group was significantly higher than that in the AF without ischemic stroke group (*P* < .05; Table 2). There were no significant differences in other parameters between the two groups.

**Table 2:** Characteristics of the included patients

Parameters	AF with ischemic stroke group (n = 132)	AF without ischemic stroke group (n = 254)	*P*
Age (y), (mean ± SD)	73.5 ± 9.3	73.1 ± 8.8	.266
Female (%)	37.9	35.0	.450
BMI (kg/m^2^), (mean ± SD)	24.5 ± 1.7	24.3 ± 2.1	.355
LncRNA ANRIL levels, (mean ± SD)	14.3 ± 2.3	11.9 ± 1.9	<.001
NIHSS at the occurrence of ischemic stroke, median (IQR)	11 (7, 17)	—	—
Infarct volume (mL), (IQR)	22 (13, 33)	—	—
Medication, (%)			
Dabigatran	22.0	25.2	.142
Rivaroxaban	23.5	15.3	
Warfarin	54.5	59.5	
mRS at 6 mo, no. (%)			
0‐2	43.2	—	—
3‐6	56.8	—	—
Total recurrence number of ischemic stroke at 6 mo, no. (%)	56.1	—	—
Hypertension (%)	37.1	42.1	.342
Diabetes mellitus (%)	16.7	15.0	.783
Smoking history (%)	65.2	46.9	<.001
Drinking history (%)	69.7	70.1	.463
Family history of ischemic stroke (%)	8.3	7.5	.718
TC (mg/dL), median (mean ± SD)	160.0 ± 26.5	155.6 ± 20.8	.165
HDL‐C (mg/dL), median (mean ± SD)	48.1 ± 7.5	50.4 ± 8.5	.089
LDL‐C (mg/dL), median (mean ± SD)	101.1 ± 24.7	98.3 ± 17.8	.155
Lp (a) (mg/L), median (mean ± SD)	172.7 ± 18.7	173.4 ± 20.7	.890
PCT (ng/mL), median (mean ± SD)	0.5 ± 0.1	0.4 ± 0.1	.231
Fibrinogen (g/L), median (mean ± SD)	2.5 ± 0.5	2.6 ± 0.7	.436
D‐dimer (mg/L), median (mean ± SD)	1.1 ± 0.3	1.0 ± 0.2	.769
Hs‐CRP (mg/dL), median (mean ± SD)	0.9 ± 0.2	0.5 ± 0.1	.265

Note

Data are percentage, (mean ± SD) or median (IQR).

Abbreviations: AF, atrial fibrillation; BMI, body mass index; HDL‐C, high‐density lipoprotein‐cholesterol; Hs‐CRP, high‐sensitivity C‐reactive protein; IQR, interquartile range; LDL‐C, low‐density lipoprotein‐cholesterol; Lp (a), lipoprotein (a); mRS, modified Rankin Scale; NIHSS, National Institutes of Health Stroke Scale; PCT, procalcitonin; TC, total cholesterol.

### 3.2 Risk factors for AF with ischemic stroke

The results of univariate and multivariate logistic regression analyses for AF with ischemic stroke are shown in Table 3. Serum lncRNA ANRIL levels and smoking were independent risk factors for AF with ischemic stroke (*P* < .05).

**Table 3:** Univariate and multivariate logistic regression analyses for AF with ischemic stroke

Parameters	Univariate analysis		Multivariate analysis^a^	
	OR (95% CI)	*P*	OR (95% CI)	*P*
Age	1.008 (0.979, 1.026)	.514		
Sex (female vs male)	0.941 (0.592, 1.813)	.879		
BMI	1.089 (0.959, 1.232)	.178		
LncRNA ANRIL	1.315 (1.024, 1.623)	<.001	1.476 (1.153, 1.879)	.002
Medication (warfarin vs dabigatran and rivaroxaban)	0.916 (0.618, 1.843)	.365		
Hypertension (yes vs no)	1.012 (0.469, 1.832)	.864		
Diabetes mellitus (yes vs no)	1.237 (0.652, 2.389)	.521		
Smoking history (yes vs no)	2.309 (1.558, 3.105)	<.001	2.018 (1.607, 2.438)	<.001
Drinking history (yes vs no)	1.536 (0.763, 1.685)	.218		
Family history of ischemic stroke (yes vs no)	1.000 (0.995, 1.008)	.708		
TC	1.003 (0.994, 1.011)	.232		
HDL‐C	0.996 (0.970, 1.027)	.875		
LDL‐C	1.003 (0.991, 1.019)	.760		
Lp (a)	0.998 (0.993, 1.008)	.512		
PCT	0.989 (0.858, 1.138)	.876		
Hs‐CRP	1.002 (0.993, 1.047)	.483		
Fibrinogen	1.110 (0.522, 2.367)	.782		
D‐dimer	0.975 (0.942, 1.008)	.115		

Abbreviation: BMI, body mass index; CI, confidence interval; HDL‐C, high‐density lipoprotein‐cholesterol; Hs‐CRP, high‐sensitivity C‐reactive protein; LDL‐C, low‐density lipoprotein‐cholesterol; Lp (a), lipoprotein (a); OR, odds ratio; PCT, procalcitonin; TC, total cholesterol.

^a^ The odds ratio of multivariate analysis was adjusted for all significant outcome predictors of the univariate logistic regression analysis.

### 3.3 The predictive value of serum lncRNA ANRIL for AF with ischemic stroke

**Figure 2:**
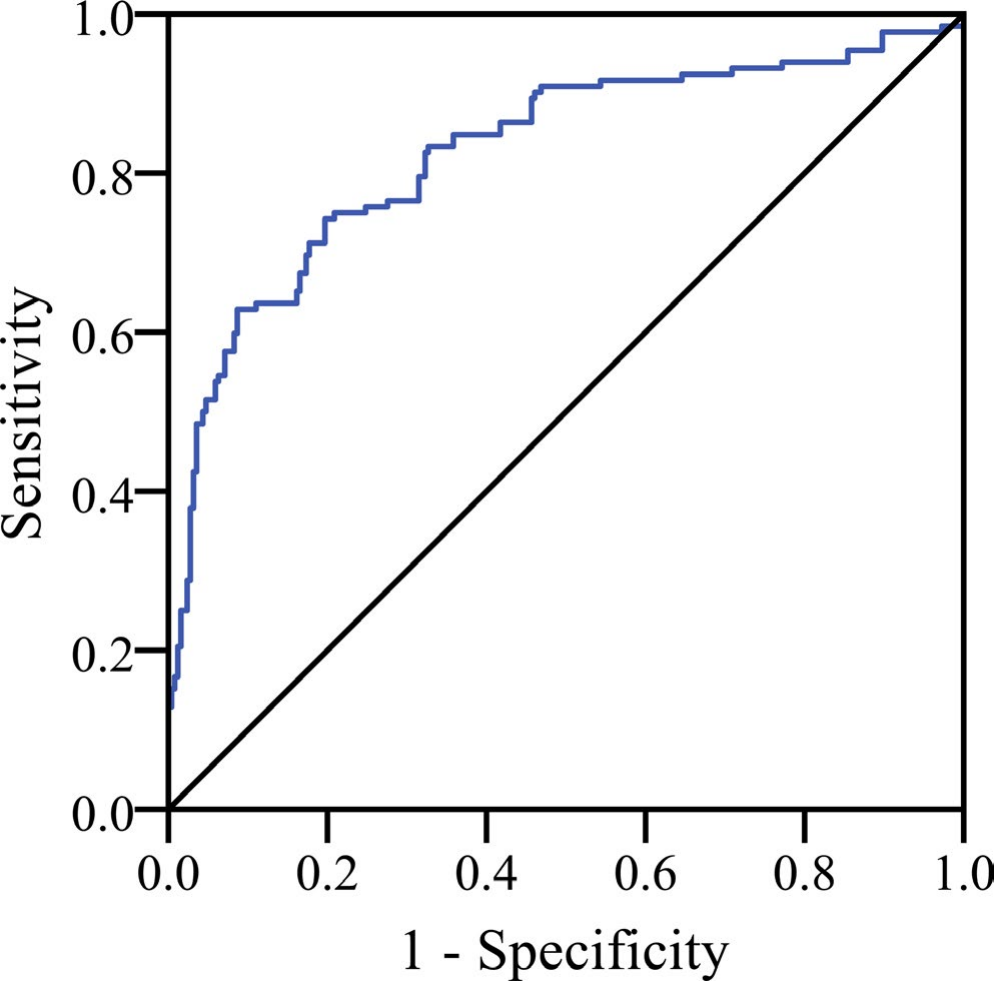
Receiver operating characteristic (ROC) curve analysis for the differential predictive value of serum lncRNA ANRIL for AF with ischemic stroke

The ROC results are shown in Figure 2. The AUC of serum lncRNA ANRIL for identifying AF with ischemic stroke was 0.826 (0.799‐0.873), the cut‐off value was 13.2, and the sensitivity and specificity were 76.6% and 81.4%, respectively.

### 3.4 Correlation of serum lncRNA ANRIL expression with NIHSS score and functional outcome

**Figure 3:**
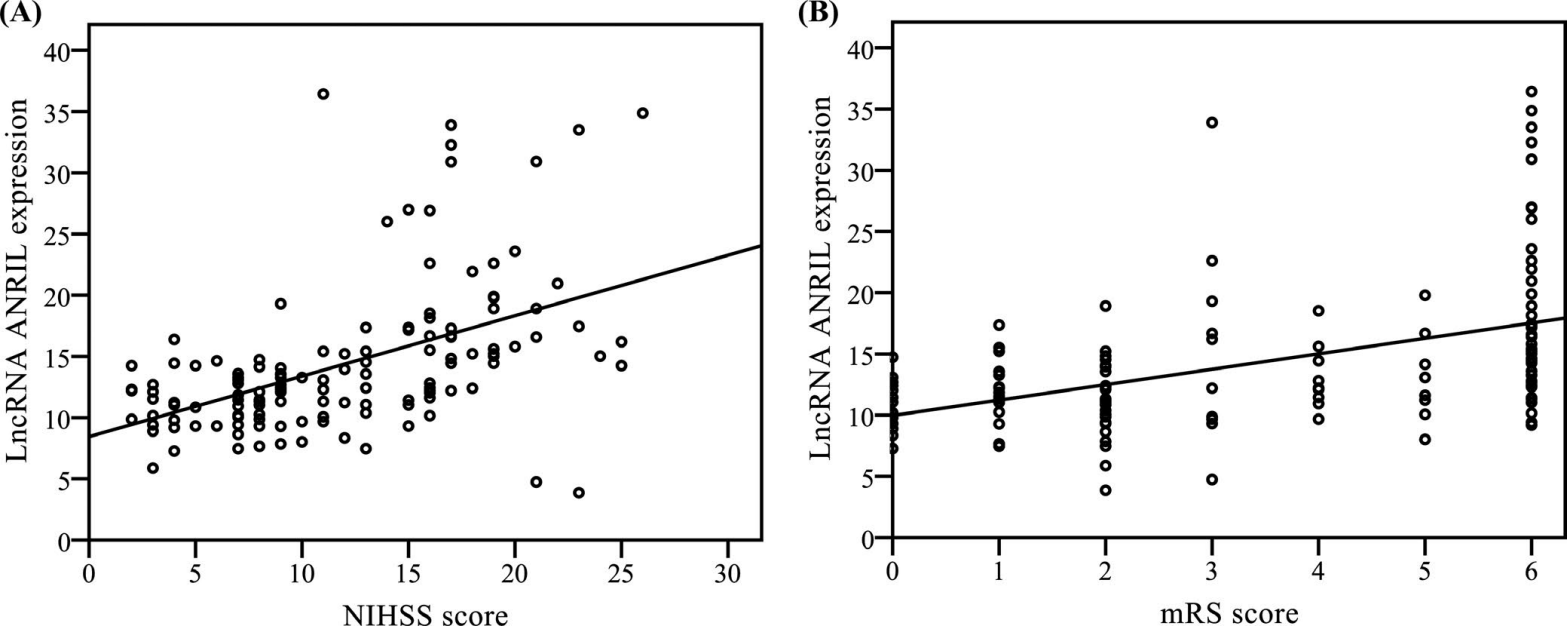
Correlation of serum lncRNA ANRIL expression with NIHSS score and functional outcome. A, Correlation between lncRNA ANRIL level and the National Institutes of Health Stroke Scale (NIHSS) score. B, Correlation between lncRNA ANRIL level and the modified Rankin Scale (mRS) score

In order to analyze the correlation between serum lncRNA ANRIL and the severity and functional outcome of patients with ischemic stroke, we analyzed the correlation of serum lncRNA ANRIL expression with NIHSS score and functional outcome. We found that serum lncRNA ANRIL was significantly correlated with the NIHSS score (*r*_Spearman_ = .490, *P* < .001; Figure 3A) and the mRS score (*r*_Spearman_ = .466, *P* < .001; Figure 3B).

### 3.5 Correlation of serum lncRNA ANRIL expression with prognosis

**Figure 4:**
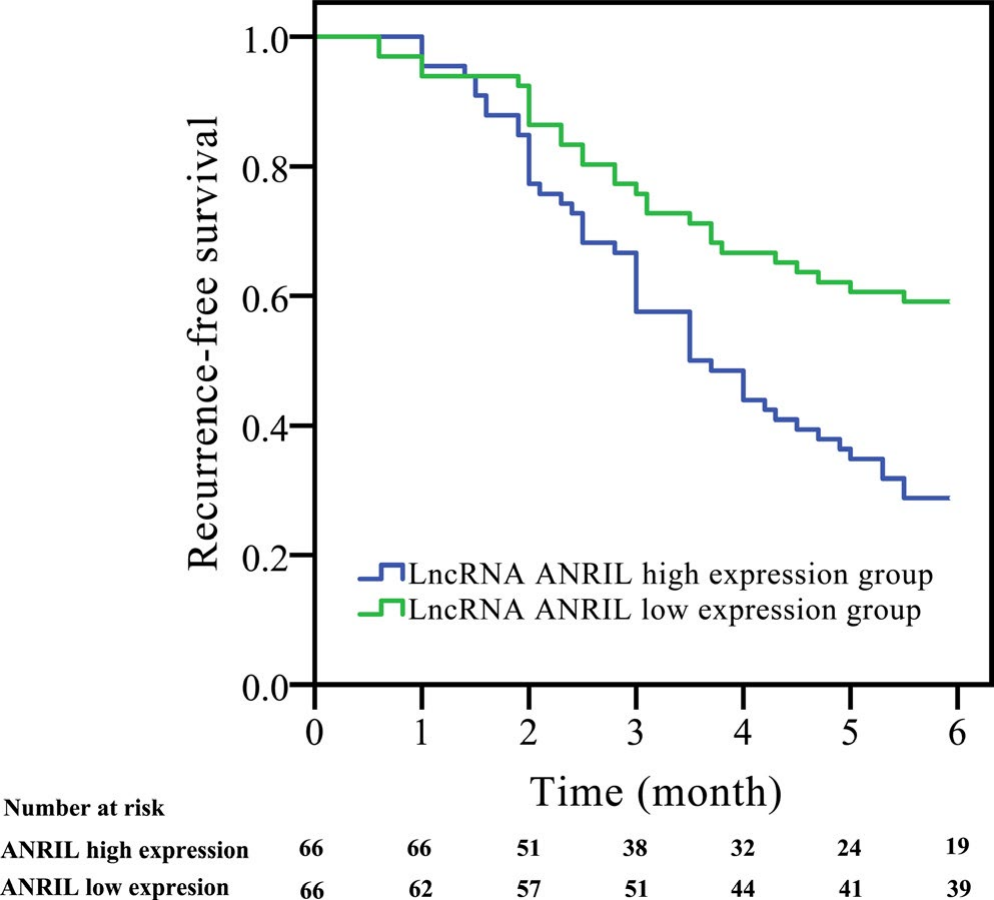
Correlation of serum lncRNA ANRIL expression with prognosis. Compared with the lncRNA ANRIL high‐expression group, the recurrence‐free survival of the lncRNA ANRIL low‐expression group was significantly higher (*χ^2^* = 11.009, log‐rank *P* < .001)

A total of 74 patients had recurrent ischemic stroke, and 48 patients died in this study. AF with ischemic stroke patients was divided into the lncRNA ANRIL high‐expression group and the lncRNA ANRIL low‐expression group according to the median serum ANRIL level. Compared with the lncRNA ANRIL high‐expression group, the recurrence‐free survival of the lncRNA ANRIL low‐expression group was significantly higher (*χ^2^* = 11.009, log‐rank *P* < .001; Figure 4). The results of Cox proportional regression model analysis of risk factors for recurrence‐free survival of AF with ischemic stroke are shown in Table 4. The serum lncRNA ANRIL level (*P* = .004), NIHSS score (*P* = .001), infarct volume (*P* = .035), and smoking (*P* < .001) were the risk factors for AF with ischemic stroke (Table 4).

**Table 4:** Cox proportional regression model analysis of risk factors for AF with ischemic stroke

Parameters	*β*	SE	Wald *χ^2^*	*P*	HR	95%*CI*
Age	.107	.072	2.225	.135	1.114	0.979 ~ 1.265
Sex (female vs male)	.233	.124	3.531	.061	1.266	0.987 ~ 1.605
BMI	.076	.101	0.547	.460	1.087	0.876 ~ 1.317
LncRNA ANRIL levels	.180	.066	7.747	.004	1.197	1.054 ~ 1.357
Medication (warfarin vs dabigatran and rivaroxaban)	.298	.316	1.537	.170	1.176	0.917 ~ 1.742
NIHSS score at the occurrence of ischemic stroke	.105	.009	136.111	.001	1.111	1.091 ~ 1.130
Infarct volume	.211	.102	4.464	.035	1.232	1.016 ~ 1.510
Hypertension (yes vs no)	.294	.173	2.888	.089	1.342	0.956 ~ 1.883
Diabetes mellitus (yes vs no)	.783	.473	2.742	.098	2.188	0.866 ~ 5.531
Smoking history (yes vs no)	.387	.026	20.520	<.001	1.472	1.251 ~ 1.738
Drinking history (yes vs no)	.632	.501	1.591	.207	1.881	0.705 ~ 5.032
Family history of ischemic stroke (yes vs no)	.725	.449	2.607	.106	2.065	0.856 ~ 4.978
TC	.377	.563	0.448	.503	1.458	0.484 ~ 4.395
HDL‐C	.286	.300	0.909	.340	1.331	0.739 ~ 2.396
LDL‐C	.198	.473	0.175	.676	1.219	0.482 ~ 3.080
Lp (a)	.273	.338	0.652	.419	1.314	0.677 ~ 2.548
PCT	.542	.302	3.221	.073	1.719	0.951 ~ 3.108
Fibrinogen	.501	.328	2.333	.127	1.650	0.868 ~ 3.139
D‐dimer	.473	.440	1.156	.282	1.605	0.677 ~ 3.802
Hs‐CRP	.732	.801	0.835	.361	2.079	0.433 ~ 9.994

Abbreviations: BMI, body mass index; *CI*, confidence interval; HDL‐C, high‐density lipoprotein‐cholesterol; HR, hazard ratio; Hs‐CRP, high‐sensitivity C‐reactive protein; LDL‐C, low‐density lipoprotein‐cholesterol; Lp (a), lipoprotein (a); mRS, modified Rankin Scale; NIHSS, National Institutes of Health Stroke Scale; PCT, procalcitonin; SE, standard error; TC, total cholesterol; *β*, regression coefficients.

## 4 DISCUSSION

LncRNAs are closely related to ischemic stroke. Dharap et al^18^ first used a rat middle cerebral artery occlusion model to evaluate the effect of ischemia on the expression of 8314 lncRNAs in the rat cerebral cortex and found that 359 expressions increased and 84 expressions decreased, indicating that cerebral ischemia significantly changed the expression of lncRNAs in the brain. After that, Zhang et al^19^ analyzed the expression of lncRNAs after 16 hours of hypoxia and glucose‐free treatment of cerebral microvascular endothelial cells and found that 147 expressions increased and 70 expressions decreased, further confirming the changes in the expression pattern of lncRNAs induced by cerebral ischemia.

LncRNA ANRIL is the best replicated genetic risk factor for coronary artery disease and regulates genes involved in fatty acid and glucose metabolism.^20^ Previous studies showed that ANRIL was highly expressed in endothelial and may be involved in the thrombogenesis.^21^, ^22^ Moreover, increased ANRIL has long been investigated as a potential marker in the plaque of atherosclerosis patients.^23^ More and more researchers began to investigate the role of ANRIL in cardiovascular diseases; however, there are little reports about the clinical and prognostic significance of ANRIL in AF with ischemic stroke.

We found that compared with the AF with ischemic stroke group, patients in the AF without ischemic stroke group had significantly lower serum lncRNA ANRIL levels. The AUC of serum lncRNA ANRIL for identifying AF with ischemic stroke was 0.826 (0.799‐0.873), and the sensitivity and specificity were 76.6% and 81.4%, respectively. Moreover, we found that serum lncRNA ANRIL was significantly correlated with the NIHSS score (*r*_Spearman_ = .490, *P* < .001) and the mRS score (*r*_Spearman_ = .466, *P* < .001). The oxidative damage induced by thrombosis in patients with AF is a hot topic in recent years. Studies have found that lncRNA ANRIL can induce thrombosis in the following ways: (a) LncRNA ANRIL can induce DNA damage in endothelial cells under oxidative stress and plays an important role in the occurrence and development of endothelial inflammation.^24^ (b) A large number of studies have pointed out that under oxidative stress, lncRNA ANRIL can promote the release of myeloperoxidase, and can induce the body to produce a variety of free radicals, promote the formation of cardiovascular plaques, and induce thrombosis.^25^ (c) In the state of oxidative stress, lncRNA ANRIL acts on the lipid peroxidation reaction product to release a large amount of malonaldehyde into peripheral blood. Malonaldehyde produces cytotoxicity by cross‐linking polymerized proteins, nucleic acids, and other macromolecules, and promotes endothelial cell damage.^26^ It is worth noting that we also found that compared with the lncRNA ANRIL high‐expression group, the recurrence‐free survival of the lncRNA ANRIL low‐expression group was significantly higher. Moreover, the serum lncRNA ANRIL level (*P* = .004), NIHSS score (*P* = .001), infarct volume (*P* = .035), and smoking (*P* < .001) were the risk factors for AF with ischemic stroke. The above results further confirm that serum lncRNA ANRIL can promote ischemic stroke in patients with AF and is closely related to patients' prognosis.

In this study, we found that serum lncRNA ANRIL has potential diagnostic and prognostic value in AF with ischemic stroke and is expected to provide new approaches and ideas for the diagnosis and treatment for AF patients.

## AUTHOR CONTRIBUTIONS

JJ conceived the study. WZ and JJ were involved in gaining ethical approval, patient recruitment, and data analysis. WZ wrote the first draft of the article. All authors reviewed and edited the article and approved the final version of the article.

## ETHICAL APPROVAL

This study was approved by the Ethics Committee of The University of Hong Kong‐Shenzhen Hospital ethics committee (LH‐S2015073).
